# Dietary fiber intake from fresh and preserved food and risk of nasopharyngeal carcinoma: observational evidence from a Chinese population

**DOI:** 10.1186/s12937-021-00667-8

**Published:** 2021-02-02

**Authors:** Zhi-Ming MAI, Roger Kai-Cheong NGAN, Dora Lai-Wan KWONG, Wai-Tong NG, Kam-Tong Yuen, Dennis Kai-Ming Ip, Yap-Hang CHAN, Anne Wing-Mui LEE, Sai-Yin HO, Maria Li LUNG, Tai-Hing LAM

**Affiliations:** 1 School of Public Health, Li Ka Shing Faculty of Medicine, The University of Hong Kong, G/F, Patrick Manson Building (North Wing), 7 Sassoon Road, Pok Fu Lam, Hong Kong, SAR China; 2 Centre for Nasopharyngeal Carcinoma Research (CNPCR), Research Grants Council Area of Excellence Scheme, The University of Hong Kong, Hong Kong, SAR China; 3 Department of Clinical Oncology, Li Ka Shing Faculty of Medicine, The University of Hong Kong, Hong Kong, SAR China; 4 Department of Clinical Oncology, Queen Elizabeth Hospital, Hong Kong, SAR China; 5 Department of Clinical Oncology, Pamela Youde Nethersole Eastern Hospital, Hong Kong, SAR China; 6 Department of Oncology, Princess Margaret Hospital, Hong Kong, SAR China; 7 Department of Medicine, Queen Mary Hospital, The University of Hong Kong, Hong Kong, SAR China; 8 Clinical Oncology Center, The University of Hong Kong-Shenzhen Hospital, Shenzhen, China

**Keywords:** Dietary fiber, Fresh vegetable and fruit, Soybean product, Preserved food, Epstein-Barr virus, Nasopharyngeal carcinoma

## Abstract

**Background:**

The role of dietary fiber intake on risk of nasopharyngeal carcinoma (NPC) remains unclear. We examined the associations of dietary fiber intake on the risk of NPC adjusting for a comprehensive list of potential confounders.

**Methods:**

Using data from a multicenter case-control study, we included 815 histologically confirmed NPC incident cases and 1502 controls in Hong Kong, China recruited in 2014–2017. Odds ratios (ORs) of NPC (cases vs controls) for dietary fiber intake from different sources at different life periods (age 13–18, age 19–30, and 10 years before recruitment) were evaluated using unconditional logistic regression, adjusting for sex, age, socioeconomic status, smoking and drinking status, occupational hazards, family history of cancer, salted fish, and total energy intake in Model 1, Epstein-Barr virus viral capsid antigen serological status in Model 2, and duration of sun exposure and circulating 25-hydroxyvitamin D in Model 3.

**Results:**

Higher intake of total dietary fiber 10 years before recruitment was significantly associated with decreased NPC risk, with demonstrable dose-response relationship (*P*-values for trend = 0.001, 0.020 and 0.024 in Models 1–3, respectively). The adjusted ORs (95% CI) in the highest versus the lowest quartile were 0.51 (0.38–0.69) in Model 1, 0.48 (0.33–0.69) in Model 2, and 0.48 (0.33–0.70) in Model 3. However, the association was less clear after adjustment of other potential confounders (e.g. EBV) in the two younger periods (age of 13–18 and 19–30 years). Risks of NPC were significantly lower for dietary fiber intake from fresh vegetables and fruits and soybean products over all three periods, with dose-response relationships observed in all Models (*P*-values for trend for age 13–18, age 19–30 and 10 years before recruitment were, respectively, 0.002, 0.009 and 0.001 for Model1; 0.020, 0.031 and 0.003 for Model 2; and 0.022, 0.037 and 0.004 for Model 3). No clear association of NPC risk with dietary fiber intake from preserved vegetables, fruits and condiments was observed.

**Conclusion:**

Our study has shown the protective role of dietary fiber from fresh food items in NPC risk, but no association for total dietary fiber intake was observed, probably because total intake also included intake of preserved food. Further studies with detailed dietary information and in prospective settings are needed to confirm this finding, and to explore the possible underlying biological mechanisms.

## Background

Fiber intake has a broad range of potential health benefits [1], including reduction in the risk of coronary heart disease [2], stroke [3], type 2 diabetes [4], colorectal cancer [5], and head and neck cancer (HNC) [6–8]. Kawakita et al. 2017 [6] conducted a pooled analysis of 10 case-control studies participating in the International Head and Neck Cancer Epidemiology consortium and reported an inverse association of fiber intake with oral and pharyngeal cancer combined, and laryngeal cancer. Their recent prospective study involving 101,700 participants also showed a potential protective role of fiber intake in the development of oral cavity and pharyngeal cancers, but no association for other types of HNC [7]. A prospective longitudinal cohort study of newly-diagnosed head and neck cancer patients showed that pretreatment dietary fiber intake was inversely associated with all-cause mortality [8]. However, these previous studies did not report the association between fiber intake and nasopharyngeal carcinoma (NPC).

NPC, a rare type of HNC in most parts of the world while relatively common in Southern China, is distinct from other types of HNC in terms of histopathological spectrum, geographical distribution, and etiology [9]. NPC age-standardized incidence rates (ASIRs) in both sexes were less than 1 per 100,000 person-years in most parts of the world [10]. However, over 60,000 new cases per year occurred in China (> 47% of all 129,000 NPCs diagnosed globally in 2018) with an ASIR of 3.0 [11]. The ASIR was particular high in south China (28.9 and 11.3 for men and women in Sihui [12], 26.8 and 10.7 in Zhongshan [13], 13.4 and 5.2 in Guangzhou [14], and 11.5 and 2.9 in Hong Kong [15]). Despite identification of several important non-viral environmental risk factors such as consumption of salted fish and preserved vegetable and fruit [16, 17], the etiology of NPC related to dietary fiber intake remains debatable. A few epidemiological studies have investigated the role of fiber-rich food intake in NPC [18–25], but only two observational studies (one case-control and one cohort study) reported protective associations of fiber intake with NPC incidence [26, 27]. However, these two studies were conducted in non-endemic populations with different dietary habits (no consumption of Chinese preserved vegetable and fruit), and they did not adjust for other important risk factors of NPC, especially Epstein-Barr virus (EBV). While EBV infection is a consistently strong risk factor of NPC and is ubiquitously present in the general populations worldwide, it cannot explain the unique geographic disease pattern of NPC [28].

We examined the association between dietary fiber intake across life periods (age 13–18, and 19–30, and 10 years before recruitment) and risk of NPC using data from the Hong Kong Area of Excellence NPC case-control (HKAoENPC) study with adjustment for a comprehensive list of potential confounders. This is a large study to examine the source-specific associations (either from fresh or preserved food items) in an NPC endemic region.

## Methods

### Study population and patient’s ascertainment

The HKAoENPC study is a multicenter case-control study conducted in five major regional hospitals (Queen Mary Hospital, Pamela Youde Nethersole Eastern Hospital, Queen Elizabeth Hospital, Princess Margaret Hospital and Tuen Mun Hospital) that treated up to 75% of all NPC new cases in Hong Kong, China. Cases and controls were recruited from March 2014 to September 2017. Detailed descriptions of the study and methods have been previously published [29, 30]. Briefly, cases were incident NPC patients diagnosed with histological and/or radiological evidence in the Department of Clinical Oncology in the hospitals (response rate 78.4%) within the past 3 months to minimize the effects of recall bias and lifestyle changes secondary to NPC. Controls were frequency-matched (by 5-year age group and sex; response rate 85.1%) new outpatient attendees or new inpatients admitted in specialist clinics of the same hospitals, within the past 12 months and 3 months, respectively. Those controls with a history of NPC, dementia, or suspected symptoms of NPC were excluded. Following the AsiaLymph guideline of the U.S. National Cancer Institute, we also specified that no more than 15% of controls had one specific type of disease. In addition to self-reported dietary information collected by the Food Frequency Questionnaires (FFQs), the subjects provided 10 ml of peripheral blood on the same date of recruitment. The samples were centrifuged at 3000 rpm at 4 degrees Celsius for 10 min, and then were stored at − 80 degrees Celsius before measurements of EBV VCA-IgA serostatus and serum level of 25-hydroxyvitamin D (25OHD).

### Dietary fiber intake

Dietary information on fiber intake was collected with the Semi-Quantitative Food Frequency Questionnaire (which was embedded in the computer-assisted, self-administered questionnaire) covering 12 high-fiber food items (6 for fresh vegetables, 3 for soybean products, and 3 for preserved foods) over three life periods (age 13–18, and 19–30, and 10 years before recruitment). We calculated dietary fiber intake by multiplying the seven categories of frequency (never, < 1 time/month, 1–2 times/month, 1–3 times/week, 4–6 times/week, 1–2 times/day, 3 times/day or more) of consumption and three categories of portion sizes (small [50 g], medium [75 g], and large [100 g], unless stated otherwise) of each item by its fiber content and summing the products across all food items in a specific period. High-fiber food groups such as fresh vegetables and fruits, soybean products, and preserved foods were assessed according to the Standard Tables of Food Composition in China (2008 No.2). For example, fresh vegetables and fruits included green leafy vegetables (lettuce, Chinese kale, Chinese flowering cabbage, Chinese white cabbage, water spinach, cabbage, broccoli, etc.), vegetables other than green leafy vegetables (cucumber, angled loofah, etc.), carrot, tomatoes, and citrus (orange, mandarin orange, pomelo, tangerine, etc.; portion: 75/150/300 g) and fruits other than citrus. Soybean products included tofu, soybean milk and bean curd. Preserved foods included items such as preserved vegetables (different types of pickled mustard, sour buckwheat head, takuan and pickled white radish), preserved fruits (salty dried plum, dried apricot, licorice olive, sun-dried tangerine peel, dried mango, preserved chayote and raisin), and condiments (shrimp paste, crab paste, soy sauce, fermented black soybean and fermented bean curd; portion: less than half of the spoon, half of the spoon, and a spoon). Participants who did not answer questions related to the frequency or consumption of food items were considered as having “zero intake.” Energy-adjustment for nutrients based on about 30 food items in our questionnaire was conducted using the residual method. Our questionnaire of food items had acceptable test-retest reliability (coefficients 0.2–0.9) [29].

### Covariate assessment

Information on covariates collected by the questionnaire included sex, 5-year age group, socioeconomic status (SES) score [range: − 1 (lowest) to 13 (highest), calculated by the subject’s, and his/her father’s and mother’s education, housing type at age 10, personal income, and household income], smoking and drinking status (ever vs never), salted fish consumption (ever vs never), occupational hazards (yes vs no), family history of cancer (yes vs no), and duration of sunlight exposure (2- < 5, 5- < 8 and 8+ vs < 2 h/day).

Antibody of IgA against EBV viral capsid antigen (VCA-IgA) was measured using a commercial kit (EUROIMMUN AG, Lübeck, Germany) based on the standard ELISA method. Results were analyzed semi-quantitatively by calculating the ratio of the optical density (OD) value of the sample over the optical OD of the calibrator, expressed as relative OD. According to the manufacturer’s instruction, the serostatus of VCA-IgA was classified as seronegative (rOD value: < 1.2) or seropositive (rOD value: ≥1.2). Serum level of 25OHD was measured using validated enzyme immunoassay (Abbott ARCHITECT i2000SR). The sensitivity was 4.75 nmol/L and the range was 0–400 nmol/L, and no sample had a concentration below or above these limits. The intra-assay coefficient of variation was 4.3–8.1% by repeating measurements of 50 samples, and the reliability coefficient was acceptable (< 10%). Circulating 25OHD was classified into three a priori categorizes based on clinically relevant cut-off points for the main analysis: < 37.5 (deficient), 37.5 < 75 (insufficient), and ≥ 75 (sufficient) nmol/L [31].

### Statistical analysis

To assess the difference between NPC cases and controls, *t*-test and *χ*^2^ test were used to compare the mean of continuous factors, and proportions of categorical factors, respectively. To examine the associations of NPC with quartiles of total dietary fiber intake, dietary fiber intake from different sources (from fresh vegetables and fruits, and soybean products, or from preserved vegetables, fruits and condiments) at each life period (age 13–18, and 19–30, and 10 years before recruitment), odds ratio (OR) and 95% confidence interval (CI) were calculated using unconditional logistic regression. The lowest quartile was set as the reference. Model 1 was adjusted for sex, 5-year age group, SES score, smoking and drinking status, occupational hazards, family history of cancer, salted fish and total energy intake as appropriate. Additional variables were adjusted for in Model 2 (EBV VCA-IgA serological status) and Model 3 (EBV VCA-IgA serological status, duration of sun exposure and circulating 25OHD). Missing values were classified as separate categories and included as indicator variables in the models. A sensitivity analysis was conducted on only World Health Organization III subtype NPC (the predominant subtype, about 90% in NPC endemic areas) [32], which did not substantially affect our results. To assess dose-response effect between exposure and NPC risk, linear trend was tested for each continuous exposure with *P*-values for trend < 0.05 indicating significance. Interactions by potential risk factors of NPC were tested based on the likelihood ratio test by introducing interaction terms into the Model 3. We repeated the analyses stratified by factors with any *P*-values for interaction < 0.2.

All statistical tests were two-sided with a specific type-I error of 0.05, and analyses were performed in Stata/SE version 15.0 software (STATA corporation, College Station, Texas, USA).

## Results

Compared with 1502 controls, the 815 NPC cases were older, had a greater proportion of men, and those with lower SES, family history of NPC, ever-smoking, EBV seropositivity, and exposure to any occupational hazards (*P*-values < 0.001). NPC cases also consumed less green leaf vegetables, other vegetables, carrot, tomato, citrus, fruits other than citrus and tofu, but more condiments than controls (*P*-values < 0.05). No difference for other factors was observed (*P* > 0.15) (Table 1).

**Table 1:** Distribution of 815 cases of NPC and 1502 controls by demographics, putative risk factors of NPC, and factors related to dietary fiber intake in Hong Kong, China, 2014–2017

Variable	NPC cases (*N* = 815)		Controls (*N* = 1502)		*P*-value‡
	n	%	n	%	
**Mean socioeconomic status score (SD)ǂ**	3	2.8	3.7	3.0	< 0.001
**Family history of cancer**					< 0.001
None	288	35.3	753	50.1	
Yes, but not NPC	269	33.0	561	37.4	
Yes, NPC	134	16.4	77	5.1	
Don’t know	124	15.2	111	7.4	
**Exposure to any occupational hazards**					< 0.001
Never	285	35.0	758	50.5	
Ever	399	49.0	597	39.8	
Don’t know	131	16.1	147	9.8	
**Smoking**					< 0.001
Never	417	51.2	945	62.9	
Ever	392	48.1	552	36.8	
Refuse to answer	6	0.7	5	0.3	
**Alcohol drinking**					0.19
Never	512	62.8	977	65.1	
≤ 210 g/week	203	24.9	377	25.1	
> 210 g/week	100	12.3	148	9.9	
**EBV VCA-IgA†**					< 0.001
Seronegative	56	10.8	902	88.1	
Seropositive	463	89.2	122	11.9	
**Sources of dietary fiber intake, g/week**					
**Fresh**					
**Green leafy vegetables**					0.001
< 9.2	327	40.1	502	33.4	
9.2- < 17.2	384	47.1	731	48.7	
17.2- < 52	93	11.4	250	16.6	
Don’t know/missing	11	1.4	19	1.3	
**Other vegetables**					0.013
< 2.2	475	58.3	831	55.3	
2.2- < 5.5	175	21.5	340	22.6	
5.5- < 24	119	14.6	377	18.4	
Don’t know/missing	46	5.6	54	3.6	
**Carrot**					< 0.001
< 0.6	318	39.0	521	34.7	
0.6- < 2.4	199	24.4	402	26.8	
2.4- < 34	132	16.2	361	24.0	
Don’t know/missing	166	20.4	218	14.5	
**Tomato**					< 0.001
< 1.9	366	44.9	618	41.2	
1.9- < 4.7	331	40.6	629	41.9	
4.7- < 40	43	5.3	156	10.4	
Don’t know/missing	75	9.2	99	6.6	
**Citrus**					0.006
< 0.6	218	26.8	344	22.9	
0.6- < 6.7	339	41.6	583	38.8	
6.7- < 29	73	9.0	189	12.6	
Don’t know/missing	185	22.7	386	25.7	
**Fruits other than citrus**					< 0.001
< 2.2	379	46.5	574	38.2	
2.2- < 3.0	216	26.5	439	29.2	
3.0- < 32	159	19.5	394	26.2	
Don’t know/missing	61	7.5	95	6.3	
**Soybean products**					
**Tofu**					0.015
< 1.2	362	44.4	603	40.2	
1.2- < 4.0	330	40.5	639	42.5	
4.0- < 51	70	8.6	182	12.1	
Don’t know/missing	53	6.5	78	5.2	
**Soymilk**					0.18
< 0.2	180	22.1	366	24.4	
0.2- < 0.4	229	28.1	437	29.1	
0.4- < 11	211	25.9	396	26.4	
Don’t know/missing	195	23.9	303	20.2	
**Bean curd**, servings/week					0.53
< 0.1	313	38.4	563	37.5	
0.1- < 0.2	181	22.2	359	23.9	
0.2- < 10	190	23.3	366	24.4	
Don’t know/missing	131	16.1	214	14.3	
**Preserved vegetables**, servings/week					0.28
< 0.5	275	33.7	512	34.1	
0.5- < 2.7	211	25.9	437	29.1	
2.7- < 44	225	27.6	385	25.6	
Don’t know/missing	104	12.8	168	11.2	
**Preserved fruits**, servings/week					0.15
< 0.3	348	42.7	666	44.3	
0.3- < 0.7	88	10.8	196	13.1	
0.7- < 33	157	19.3	285	19.0	
Don’t know/missing	222	27.2	355	23.6	
**Condiments**, servings/week					0.022
< 0.5	260	31.9	552	36.8	
0.5- < 2.2	283	34.7	456	30.4	
2.2- < 24	211	25.9	408	27.2	
Don’t know/missing	61	7.5	86	5.7	

Abbreviation: *NPC* Nasopharyngeal carcinoma; *SD* Standard deviation; *g* Gram; EBV VCA-IgA, IgA against Epstein-Barr virus viral capsid antigen VCA

‡ t-test and Chi-square test were used to compare the mean of continuous factors, and proportions of categorical factors between cases and controls, respectively

**ǂ** Socioeconomic status score ranged from −1 (lowest socioeconomic status) to 13 (highest socioeconomic status), and was calculated by the subject’s, and his/her father’s and mother’s education, personal income, household income and housing type at age 10

† Epstein-Barr virus viral capsid antibody (EBV VCA-IgA) levels: optical density value < 1.2 (seronegative) or ≥ 1.2 (seropositive)

Higher intake of total dietary fiber 10 years before recruitment was significantly associated with decreased NPC risk, with demonstrable dose-response relationship (*P*-values for trend = 0.001, 0.020 and 0.024 in Models 1–3, respectively). The adjusted ORs (95% CI) in the highest versus the lowest quartile were 0.51 (0.38–0.69) in Model 1, 0.48 (0.33–0.69) in Model 2, and 0.48 (0.33–0.70) in Model 3. While higher intake of total dietary fiber both at age of 13–18 and 19–30 years was also significantly associated with decreased NPC risk with dose-response relationships in Model 1 (OR in the highest versus the lowest quartile = 0.56, 95% CI = 0.40–0.79, *P* for trend = 0.006, and 0.49, 0.37–0.66, 0.003, respectively), these dose-response relationships became non-significant after additionally adjusting for EBV VCA-IgA in Model 2, and sun exposure and 25OHD in Model 3. Risks of NPC were also significantly lower for dietary fiber intake from fresh vegetables and fruits and soybean products over all three periods, with dose-response relationships (*P*-values for trend = 0.002 for age of 13–18 years, 0.009 for age of 19–30 years and 0.001 for 10 years before recruitment, 0.020, 0.031 and 0.003, and 0.022, 0.037 and 0.004 in Models 1–3, respectively) observed in all Models. No clear association of NPC risk with dietary fiber intake from preserved vegetables, fruits and condiments was observed (Table 2).

**Table 2:** Adjusted† OR and 95% CI of NPC with dietary fiber intake in Hong Kong, China, 2014–2017

			Model 1					Model 2					Model 3				
			Quartiles of intake OR 95% CI				*P* for trend‡	Quartiles of intake OR 95% CI				*P* for trend‡	Quartiles of intake OR 95% CI				*P* for trend‡
**All subjects**	Mean	SD															
	(g/week)		1 (lowest)	2	3	4 (highest)		1 (lowest)	2	3	4 (highest)		1 (lowest)	2	3	4 (highest)	
**Total fiber intake**																	
*age of 13–18 years*	29.0	18.1	1 (Ref)	**0.86**	**0.75**	**0.56**	0.006	1 (Ref)	**1.05**	**0.86**	**0.55**	0.07	1 (Ref)	**1.06**	**0.86**	**0.55**	0.08
				0.65–1.13	0.56–1.00	0.40–0.79			0.74–1.50	0.59–1.24	0.35–0.84			0.74–1.51	0.59–1.24	0.36–0.86	
*age of 19–30 years*	30.6	19.0	1 (Ref)	**0.82**	**0.65**	**0.49**	0.003	1 (Ref)	**1.02**	**0.75**	**0.49**	0.05	1 (Ref)	**1.01**	**0.75**	**0.49**	0.06
				0.64–1.05	0.50–0.85	0.37–0.66			0.75–1.38	0.54–1.04	0.34–0.71			0.74–1.37	0.54–1.05	0.34–0.71	
*10 years before recruitment*	31.5	19.0	1 (Ref)	**0.83**	**0.66**	**0.51**	0.001	1 (Ref)	**0.91**	**0.73**	**0.48**	0.020	1 (Ref)	**0.91**	**0.73**	**0.48**	0.024
				0.65–1.06	0.51–0.86	0.38–0.69			0.67–1.24	0.53–1.01	0.33–0.69			0.69–1.23	0.53–1.02	0.33–0.70	
**Sources**																	
**From intake of fresh vegetables and fruits and soybean products**																	
*age of 13–18 years*	25.1	15.8	1 (Ref)	**0.89**	**0.73**	**0.59**	0.002	1 (Ref)	**1.02**	**0.77**	**0.59**	0.020	1 (Ref)	**1.02**	**0.75**	**0.59**	0.022
				0.67–1.18	0.55–0.97	0.42–0.82			0.72–1.46	0.54–1.10	0.39–0.89			0.71–1.46	0.53–1.08	0.39–0.90	
*age of 19–30 years*	26.8	16.8	1 (Ref)	**1.08**	**0.71**	**0.64**	0.009	1 (Ref)	**1.24**	**0.77**	**0.55**	0.031	1 (Ref)	**1.22**	**0.76**	**0.55**	0.037
				0.83–1.42	0.53–0.94	0.46–0.89			0.88–1.76	0.53–1.10	0.36–0.83			0.86–1.73	0.53–1.09	0.36–0.83	
*10 years before recruitment*	28.0	17.0	1 (Ref)	**0.93**	**0.62**	**0.49**	0.001	1 (Ref)	**1.02**	**0.66**	**0.41**	0.003	1 (Ref)	**1.01**	**0.65**	**0.41**	0.004
				0.71–1.22	0.46–0.82	0.35–0.68			0.72–1.44	0.46–0.95	0.27–0.62			0.71–1.42	0.45–0.94	0.27–0.63	
**From intake of preserved vegetables, fruits and condiments**																	
*age of 13–18 years*	3.9	5.2	1 (Ref)	**1.02**	**1.11**	**1.16**	0.33	1 (Ref)	**0.92**	**1.38**	**1.33**	0.13	1 (Ref)	**0.91**	**1.38**	**1.34**	0.13
				0.76–1.37	0.83–1.49	0.84–1.60			0.64–1.34	0.95–1.99	0.88–2.01			0.62–1.31	0.95–2.00	0.89–2.04	
*age of 19–30 years*	3.8	5.2	1 (Ref)	**1.14**	**1.31**	**1.01**	0.74	1 (Ref)	**1.05**	**1.53**	**1.10**	0.83	1 (Ref)	**1.04**	**1.54**	**1.12**	0.82
				0.87–1.49	1.01–1.70	0.76–1.34			0.75–1.46	1.11–2.12	0.77–1.57			0.75–1.46	1.11–2.13	0.78–1.60	
*10 years before recruitment*	3.5	4.8	1 (Ref)	**1.24**	**1.39**	**1.12**	0.92	1 (Ref)	**1.25**	**1.57**	**1.26**	1.00	1 (Ref)	**1.24**	**1.57**	**1.28**	0.99
				0.95–1.62	1.07–1.81	0.85–1.49			0.90–1.74	1.13–2.19	0.88–1.80			0.89–1.73	1.13–2.18	0.89–1.83	

Abbreviation: *OR* Odds ratio; *CI* Confidence interval; *SD* Standard deviation; *g* Gram; *ref*. Reference group; EBV VCA-IgA, IgA against Epstein-Barr virus viral capsid antigen VCA

† Model 1: adjusted for sex and 5-year age group (frequency match), and socioeconomic status score (range: −1 [lowest] to 13 [highest], calculated by the subject’s, and his/her father’s and mother’s education, housing type at age 10, personal income and household income), smoking and drinking status (never/ever), occupational hazards (never/ever), family history of cancer (none/NPC/other cancers), consumption of salted fish (never/ever), and total energy intake (residual method) as appropriate

Model 2: Model 1 + EBV VCA-IgA (seronegative/seropositive)

Model 3: Model 1 + EBV VCA-IgA (seronegative/seropositive) + duration of sun exposure (< 2/2- < 5/5- < 8/8h hours/day) and circulating levels of 25-hydroxyvitamin D (< 37.5/37.5 < 75/≥75 nmol/L)

‡ Test for trend was examined for a model that included continuous fiber variables

No evidence (*P*-values for interaction > 0.20) of interaction was found by sex, age, drinking status, occupational hazards, family history of cancer, consumption of salted fish, duration of sun exposure and circulating levels of 25-hydroxyvitamin D for the association between dietary fiber intake and NPC, except for SES and tobacco use. Higher NPC risks were observed for the highest quartile of dietary fiber intake from preserved vegetables, fruits and condiments 10 years before recruitment in participants with high SES (adjusted OR = 2.30, 95% CI = 1.26–4.19), but not with low SES (0.92–0.58-1.45) (*P* for interaction = 0.067). In addition, risks of NPC were significantly lower for higher total dietary fiber intake or intake from fresh vegetables and fruits and soybean products 10 years before recruitment only in never smokers, but not in ever smokers. The *P*-values for trend were 0.025 and 0.009 in never smokers, and 0.27 and 0.41 in ever smokers (*P*-values for interaction = 0.112 and 0.189, respectively) (Table 3).

**Table 3:** Adjusted† OR and 95% CI of NPC with dietary fiber intake by socioeconomic status and tobacco use in Hong Kong China, 2014–2017

			Low SES^⋁^					High SES^⋁^			*P* for interaction
	OR 95% CI				*P* for trend‡	OR 95% CI				*P* for trend‡	
**All subjects**											
	1 (lowest)	2	3	4 (highest)		1 (lowest)	2	3	4 (highest)		
**Total fiber intake**											
*10 years before recruitment*	1 (Ref)	**0.88**	**0.78**	**0.46**	0.16	1 (Ref)	**0.93**	**0.67**	**0.51**	0.07	0.79
		0.59–1.30	0.51–1.20	0.28–0.74			0.56–1.55	0.40–1.11	0.28–0.94		
**Sources**											
**From intake of fresh vegetables and fruits and soybean products**											
*10 years before recruitment*	1 (Ref)	**0.92**	**0.73**	**0.52**	0.27	1 (Ref)	**0.92**	**0.66**	**0.35**	0.011	0.61
		0.62–1.35	0.48–1.11	0.33–0.85			0.56–1.52	0.39–1.12	0.19–0.64		
**From intake of preserved vegetables, fruits and condiments**											
*10 years before recruitment*	1 (Ref)	**1.19**	**1.29**	**0.92**	0.20	1 (Ref)	**1.51**	**2.21**	**2.30**	0.11	**0.07**
		0.78–1.83	0.84–1.97	0.58–1.45			0.87–2.62	1.27–3.85	1.26–4.19		
	**Never smokers**					**Ever smokers**					
	OR 95% CI				*P* for trend‡	OR 95% CI				*P* for trend‡	
**All subjects**											
	1 (lowest)	2	3	4 (highest)		1 (lowest)	2	3	4 (highest)		
**Total fiber intake**											
*10 years before recruitment*	1 (Ref)	**0.73**	**0.53**	**0.42**	0.025	1 (Ref)	**1.13**	**1.05**	**0.48**	0.27	**0.11**
		0.47–1.13	0.34–0.82	0.26–0.70			0.72–1.78	0.63–1.74	0.27–0.86		
**Sources**											
**From intake of fresh vegetable and fruit and soybean product**											
*10 years before recruitment*	1 (Ref)	**0.78**	**0.50**	**0.35**	0.009	1 (Ref)	**1.09**	**1.02**	**0.51**	0.41	**0.19**
		0.51–1.19	0.32–0.77	0.22–0.58			0.70–1.69	0.62–1.68	0.28–0.92		
**From intake of preserved vegetable, fruit and condiment**											
*10 years before recruitment*	1 (Ref)	**1.32**	**1.69**	**1.72**	0.36	1 (Ref)	**1.18**	**1.47**	**0.95**	0.28	0.60
		0.84–2.07	1.08–2.64	1.05–2.80			0.71–1.98	0.88–2.47	0.55–1.62		

Abbreviation: *OR* Odds ratio; *CI* Confidence interval; *SES* Socioeconomic status score; *g* Gram; *ref*. Reference group; EBV VCA-IgA, IgA against Epstein-Barr virus viral capsid antigen VCA

† Adjusted for sex and 5-year age group (frequency match), and SES score (range: −1 [lowest] to 13 [highest], calculated by the subject’s, and his/her father’s and mother’s education, housing type at age 10, personal income and household income), smoking and drinking status (never/ever), occupational hazards (never/ever), family history of cancer (none/NPC/other cancers), consumption of salted fish (never/ever), and total energy intake (residual method), EBV VCA-IgA (seronegative/seropositive), duration of sun exposure (< 2/2- < 5/5- < 8/8h hours/day) and circulating levels of 25-hydroxyvitamin D (< 37.5/37.5 < 75/≥75 nmol/L)

^⋁^ Low SES: SES score < 4; High SES: SES score ≥ 4

‡ Test for trend was examined for a model that included continuous fiber variables

*P*-values for interactions by sex, age (< 40, 40- < 65, 65+), drinking status (never, ever), occupational hazards (never/ever), family history of cancer (none/NPC/other cancers), consumption of salted fish (never/ever), duration of sun exposure (< 5/5h hours/day) and circulating levels of 25-hydroxyvitamin D (< 37.5/37.5 < 75/≥75 nmol/L) for the association between dietary fiber intake and NPC ranged from 0.22–0.99, except for SES and tobacco use (*P* for interaction < 0.20)

## Discussion

Our study has shown an inverse association between total dietary fiber intake 10 years before recruitment and NPC risk in Hong Kong, where NPC is endemic. The association was less clear after adjustment of other potential confounders (e.g. EBV) in the two younger periods (age of 13–18 and 19–30 years). Our results can strengthen the evidence for dietary fiber intake from fresh foods as a protective factor of NPC, which were consistent across life periods, but we found no evidence for an association for dietary fiber intake from preserved foods. We also observed a multiplicative interaction (marginal *P*-values < 0.20) with SES and smoking for the association between fiber intake and NPC risk. The combination of high SES & high dietary fiber intake from preserved foods was associated with a higher excess risk of NPC, whereas the protective role of dietary fiber intake (total or from fresh foods) was only observed in never smokers.

These findings are consistent with results from one case-control study and one prospective cohort study both in NPC non-endemic regions [26, 27]. An Italian case-control study by Bidoli et al. on 198 NPC cases and 594 non-NPC controls during 1992 to 2008 using total fiber intake based on a 78-item food frequency questionnaire found a suggestive inverse association between total fiber intake and NPC risk (OR = 0.58 in the highest versus the lowest quartile of intake, 95% CI = 0.34–0.96) [26]. A prospective analysis by Kasum et al. on 18 NPC cases in 34,351 postmenopausal women in the Iowa Women’s Health Cohort Study in the United States found a non-significant inverse association between total fiber intake and NPC risk [27]. Both studies did not account for potential confounders (e.g. EBV) and fiber intake from preserved foods, and did not examine potential interactions by other putative risk factors. Epidemiological studies from NPC endemic regions have shown inverse associations of NPC risk with consumption of fresh vegetables and fruits [18–25], and our findings are somewhat in agreement with these results. However, these studies did not calculate total dietary fiber intake.

While further prospective studies in other populations are needed to confirm this finding, several biological mechanisms have been proposed to explain the protective role of fiber intake in NPC. One possibility is that fiber is known to have anti-inflammatory effects [33, 34]. Another possibility is that a high fiber intake may simply be an indicator of a healthier lifestyle (e.g. never smoked) [35]. Indeed, our results from stratified analysis by tobacco use support this hypothesis, as total dietary fiber intake and intake from fresh foods were significantly associated with lower risk of NPC in never smokers only.

A strength of our study was that it has included a comprehensive list of potential confounders, and is the first to show the role of dietary fiber intake from fresh and preserved food items in an NPC endemic region. The limitations of our study are common to those that use FFQs to estimate dietary intakes [36]. The estimation of intake of dietary fiber using FFQs may have considerable error. In addition, as a hospital-based case-control study, Berkson’s bias, recall bias and residual confounding could be present. Because we used incident cases in our case-control study, indirect Berkson’s bias (exposure-disease associations that arise because another disease is associated with the exposure under study) is unlikely [37]. The intake of dietary fiber in our control group was lower than that in the general Hong Kong population [38], suggesting that our results might have underestimated the protective role of fiber intake in NPC. However, recruiting healthy controls from the community would not be the best method due to recall bias from healthy subjects (versus NPC and other hospital patients). Our hospital patients had more similar mindset and recall behavior with NPC patients than community controls, resulting in less subjective bias. We conducted a test-retest reliability study to assess recall error, and found that the questionnaire data of most NPC etiology factors of our NPC cases and controls had acceptable reliability [29]. While we had adjusted for the most relevant and potential confounders, residual confounding cannot be ruled out. The association between dietary fiber and NPC risk could have been underestimated because the dietary variables such as rice and wheat were imperfectly measured in our questionnaire.

## Conclusion

Our study has shown the protective role of dietary fiber from fresh food items in NPC risk, but no association for total dietary fiber intake was observed, probably because total intake also included intake of preserved food. Our results suggest that increased fiber intake from fresh vegetables, fruits, and soybean products but not preserved vegetables, fruits, and condiments could be an option for achieving an adequate fiber intake for the prevention of NPC. Further studies with detailed dietary information and in prospective settings are needed to confirm this finding, and to explore the possible underlying biological mechanisms.

## Data Availability

The datasets used and/or analyzed during the current study are available from the corresponding author on reasonable request.

## References

[1] Reynolds A, Mann J, Cummings J, Winter N, Mete E, Te Morenga L (2019). Carbohydrate quality and human health: a series of systematic reviews and meta-analyses. Lancet.

[2] Hartley L, May MD, Loveman E, Colquitt JL, Rees K. Dietary fibre for the primary prevention of cardiovascular disease. Cochrane Database Syst Rev. 2016;(1):CD011472. 10.1002/14651858.CD011472.pub2. 10.1002/14651858.CD011472.pub2 PMC7032538 26758499

[3] Threapleton DE, Greenwood DC, Evans CE, Cleghorn CL, Nykjaer C, Woodhead C, Cade JE, Gale CP, Burley VJ (2013). Dietary fiber intake and risk of first stroke: a systematic review and meta-analysis. Stroke.

[4] Silva FM, Kramer CK, de Almeida JC, Steemburgo T, Gross JL, Azevedo MJ (2013). Fiber intake and glycemic control in patients with type 2 diabetes mellitus: a systematic review with meta-analysis of randomized controlled trials. Nutr Rev.

[5] World Cancer Research Fund/American Institute for Cancer Research. Continuous Update Project Expert Report 2018. Diet, nutrition, physical activity and colorectal cancer. Available at dietandcancerreport.org.

[6] Kawakita D, Lee YCA, Turati F, Parpinel M, Decarli A, Serraino D, Matsuo K, Olshan AF, Zevallos JP, Winn DM (2017). Dietary fiber intake and head and neck cancer risk: a pooled analysis in the international head and neck Cancer epidemiology consortium. Int J Cancer.

[7] Kawakita D, Lee YCA, Gren LH, Buys SS, La Vecchia C, Hashibe M (2019). Fiber intake and the risk of head and neck cancer in the prostate, lung, colorectal and ovarian (PLCO) cohort. Int J Cancer.

[8] Maino Vieytes CA, Mondul AM, Li Z, Zarins KR, Wolf GT, Rozek LS, Arthur AE (2019). Dietary Fiber, whole grains, and head and neck Cancer prognosis: findings from a prospective cohort study. Nutrients.

[9] Lee AW, Lung ML, Ng WT. Nasopharyngeal carcinoma: from etiology to clinical practice. Lodon: Academic Press; 2019.

[10] Chen YP, Chan AT, Le QT, Blanchard P, Sun Y, Ma J. Nasopharyngeal carcinoma. Lancet. 2019;394:64–80. 10.1016/S0140-6736(19)30956-0 31178151

[11] Ferlay J, Soerjomataram I, Ervik M (2013). GLOBOCAN 2012 v1.0, Cancer incidence and mortality worldwide: IARC CancerBase no. 11.

[12] Zhang LF, Li YH, Xie SH, Ling W, Chen SH, Liu Q, Huang QH, Cao SM. Incidence trend of nasopharyngeal carcinoma from 1987 to 2011 in Sihui County, Guangdong Province, South China: an age-period-cohort analysis. Chin J Cancer. 2015;34:15. 10.1186/s40880-015-0018-6 PMC4593377 26058679

[13] Tang LL, Chen WQ, Xue WQ, He YQ, Zheng RS, Zeng YX, Jia WH. Global trends in incidence and mortality of nasopharyngeal carcinoma. Cancer Lett. 2016;374:22–30. 10.1016/j.canlet.2016.01.040 26828135

[14] Li K, Lin GZ, Shen JC, Zhou Q (2014). Time trends of nasopharyngeal carcinoma in urban Guangzhou over a 12-year period (2000-2011): declines in both incidence and mortality. Asian Pac J Cancer Prev.

[15] Hong Kong Cancer Registry. Nasopharyngeal Cancer in 2018. Hong Kong Hospital Authority. 2020. Available at: https://www3.ha.org.hk/cancereg/allages.asp. Accessed 24 Dec 2020.

[16] Chang ET, Adami H-O (2006). The enigmatic epidemiology of nasopharyngeal carcinoma. Cancer Epidemiol Biomark Prev.

[17] Jia WH, Qin HD. Non-viral environmental risk factors for nasopharyngeal carcinoma: a systematic review. Semin Cancer Biol. 2012;22:117–26. 10.1016/j.semcancer.2012.01.009 22311401

[18] Yu MC, Huang TB, Henderson BE (1989). Diet and nasopharyngeal carcinoma: a case-control study in Guangzhou, China. Int J Cancer.

[19] Zheng Y, Tuppin P, Hubert A, Jeannel D, Pan Y, Zeng Y, De Thé G (1994). Environmental and dietary risk factors for nasopharyngeal carcinoma: a case-control study in Zangwu County, Guangxi, China. Br J Cancer.

[20] Gallicchio L, Matanoski G, Tao X, Chen L, Lam TK, Boyd K, Robinson KA, Balick L, Mickelson S, Caulfield LE (2006). Adulthood consumption of preserved and nonpreserved vegetables and the risk of nasopharyngeal carcinoma: a systematic review. Int J Cancer.

[21] Jia WH, Luo XY, Feng BJ, Ruan HL, Bei JX, Liu WS, Qin HD, Feng QS, Chen LZ, Yao SY. Traditional Cantonese diet and nasopharyngeal carcinoma risk: a large-scale case-control study in Guangdong, China. BMC Cancer. 2010;10:1–7. 10.1186/1471-2407-10-446 PMC2931495 20727127

[22] Liu YT, Dai JJ, Xu CH, Lu YK, Fan YY, Zhang XL, Zhang CX, Chen YM. Greater intake of fruit and vegetables is associated with lower risk of nasopharyngeal carcinoma in Chinese adults: a case–control study. Cancer Causes Control. 2012;23:589–99. 10.1007/s10552-012-9923-z 22392078

[23] Polesel J, Serraino D, Negri E, Barzan L, Vaccher E, Montella M, Zucchetto A, Garavello W, Franceschi S, La Vecchia C (2013). Consumption of fruit, vegetables, and other food groups and the risk of nasopharyngeal carcinoma. Cancer Causes Control.

[24] Feng XX, Wang MX, Li M, Tang X, Jiang H, Wang R, Ma L, Yin Y, Wu CR. Citrus fruit intake and the risk of nasopharyngeal carcinoma. Asia Pac J Clin Nutr. 2019;28:783. 10.6133/apjcn.201912_28(4).0015 31826376

[25] Jin J, Ouyang Z, Wang Z (2014). Association of fruit and vegetables with the risk of nasopharyngeal cancer: evidence from a meta-analysis. Sci Rep.

[26] Bidoli E, Pelucchi C, Polesel J, Negri E, Barzan L, Franchin G, Franceschi S, Serraino D, Paoli PD, Vecchia CL (2013). Fiber intake and risk of nasopharyngeal carcinoma: a case-control study. Nutrition Cancer.

[27] Kasum CM, Jacobs DR, Nicodemus K, Folsom AR (2002). Dietary risk factors for upper aerodigestive tract cancers. Int J Cancer.

[28] Lee AW, Ng W, Chan Y, Sze H, Chan C, Lam T. The battle against nasopharyngeal cancer. Radiother Oncol. 2012. 10.1016/j.radonc.2012.08.001 22938727

[29] Mai ZM, Lin JH, Chiang SC, Ngan RKC, Kwong DLW, Ng WT, Ng AWY, Yuen KT, Ip KM, Chan YH, Lee AWM, Ho SY, Lung ML, Lam TH. Test-retest reliability of a computer-assisted self-administered questionnaire on early life exposure in a nasopharyngeal carcinoma case-control study. Sci Rep. 2018;8:1–7. 10.1038/s41598-018-25046-y PMC5935670 29728581

[30] Mai ZM, Lin JH, Ngan RKC, Kwong DLW, Ng WT, Ng AWY, Yuen KT, Ip DKM, Chan YH, Lee AWM, Ho SY, Lung ML, Lam TH. Milk consumption across life periods in relation to lower risk of nasopharyngeal carcinoma: a multicentre case-control study. Front Oncol. 2019;9:253. 10.3389/fonc.2019.00253 PMC6467951 31024854

[31] Mai ZM, Lin JH, Ngan RKC, Kwong DLW, Ng WT, Ng AWY, Ip KM, Chan YH, Lee AWM, Ho SY, Lung ML, Lam TH. Solar ultraviolet radiation and vitamin D deficiency on Epstein-Barr virus reactivation: observational and genetic evidence from a nasopharyngeal carcinoma endemic population. Open Forum Infect Dis. 2020. 10.1093/ofid/ofaa426 PMC7585328 33134413

[32] Tse L, Yu IT, Mang OW, Wong S (2006). Incidence rate trends of histological subtypes of nasopharyngeal carcinoma in Hong Kong. Br J Cancer.

[33] Ma Y, Hébert JR, Li W, Bertone-Johnson ER, Olendzki B, Pagoto SL, Tinker L, Rosal MC, Ockene IS, Ockene JK (2008). Association between dietary fiber and markers of systemic inflammation in the Women's Health Initiative observational study. Nutrition.

[34] Krishnamurthy VMR, Wei G, Baird BC, Murtaugh M, Chonchol MB, Raphael KL, Greene T, Beddhu S (2012). High dietary fiber intake is associated with decreased inflammation and all-cause mortality in patients with chronic kidney disease. Kidney Int.

[35] Bosetti C, Pelucchi C, La Vecchia C (2009). Diet and cancer in Mediterranean countries: carbohydrates and fats. Public Health Nutr.

[36] Willett W. Nutritional epidemiology. Oxford: Oxford university press; 2012. p.7–12.

[37] Berkson J (2014). Limitations of the application of fourfold table analysis to hospital data. Int J Epidemiol.

[38] Report of Health Behaviour Survey 2018/19. Hong Kong: non-communicable disease branch, Centre for health protection, Department of Health; 2020.

